# Ionotropic Glutamate Receptor Function in Interpeduncular Nucleus Is Modulated by Nicotine Exposure

**DOI:** 10.1523/ENEURO.0052-25.2025

**Published:** 2025-12-02

**Authors:** Yijin Yan, Brenton R. Tucker, Andrew E. Tapp, Leanne N. Thomas, Dylan R. Drenan, Ryan M. Drenan

**Affiliations:** Department of Translational Neuroscience, Wake Forest University School of Medicine, Winston-Salem, North Carolina 27101

**Keywords:** addiction, glutamate, nicotine, receptor, self-administration, uncaging

## Abstract

The medial habenula (MHb) and its main projection target, the interpeduncular nucleus (IPN), play an important role in mood/affect, anxiety, and the aversive experience associated with nicotine withdrawal. Given that MHb axons release glutamate onto IPN neurons, we investigated the expression and functional responses of ionotropic glutamate receptors (iGluRs) in neurons of the rostral IPN (IPR) in male rats. After confirming mRNA expression of *Gria1* and *Grin1* iGluR subunits in IPR, we employed glutamate uncaging coupled with two-photon imaging and patch-clamp electrophysiology. IPR dendrites, which often contained spine-like protrusions suggestive of synaptic contacts, featured a variety of response profiles following localized glutamate uncaging. Pharmacology experiments confirmed functional α-amino-3-hydroxy-5-methyl-4-isoxazolepropionic acid and *N*-methyl-d-aspartate iGluR responses in IPR neuronal somata. Rats were trained to self-administer nicotine or saline during 10 fixed ratio 1 sessions and seven intermittent access sessions. In rats with a history of nicotine self-administration, perisomatic IPR iGluR responses are reduced. Acute nicotine application to slices from drug-naive rats recapitulated the effect of nicotine self-administration. These results identify a mechanism, whereby nicotine, even acute nicotine, may reduce IPR neuron sensitivity to glutamate from MHb axons, which could play a role in the aversive response to nicotine withdrawal.

## Significance Statement

The medial habenula to interpeduncular nucleus (IPN) pathway plays a key role in withdrawal responses following nicotine discontinuation, and glutamatergic transmission is required for this. This study examines ionotropic glutamate receptor (iGluR) expression, function, and subcellular distribution in IPN neurons of naive rats and rats with a history of nicotine self-administration. Acute or chronic nicotine exposure alters iGluR function in these cells, suggesting that nicotine will dynamically modulate the excitability of IPN neurons in tobacco users. These results add to our growing understanding of the cellular, circuit, and pharmacological mechanisms giving rise to nicotine dependence.

## Introduction

The medial habenula (MHb) is a small epithalamic nucleus connecting the limbic forebrain to midbrain/hindbrain neuromodulatory nuclei via its projection to the interpeduncular nucleus (IPN). In animal models, discontinuation of nicotine exposure elicits withdrawal-like behaviors via the MHb–IPN circuit and the nicotinic acetylcholine receptors expressed therein (Perry et al., 2002; Salas et al., 2004; Grady et al., 2009; Shih et al., 2014). Human mutations in a nAChR gene cluster encoding α3, β4, and α5 subunits—which are strongly and selectively expressed in the MHb—IPN circuit (Shih et al., 2014)—are associated with nicotine dependence and lung cancer (Bierut et al., 2008; Wang et al., 2009). In rodent models, Kenny (Fowler et al., 2011), Maskos (Forget et al., 2018), and others (Frahm et al., 2011) have demonstrated that α5-containing nAChRs transmit signals related to nicotine aversion. Several groups (Hsu et al., 2013; Ables et al., 2017; Arvin et al., 2019) have shown that α5 nAChR expression is enriched in the IPN—specifically the rostral subnucleus (the IPR).

The ventral MHb is a dense cluster of neurons expressing biosynthetic machinery for both acetylcholine (Sheffield et al., 2000; Ren et al., 2011; Aizawa et al., 2012; Frahm et al., 2015) and glutamate (Ren et al., 2011; Aizawa et al., 2012; Frahm et al., 2015; Ables et al., 2017). They express exquisitely high levels of nAChRs (Quick et al., 1999; Shih et al., 2014; Arvin et al., 2019), yet they do not appear to receive afferent cholinergic inputs (Chung et al., 2023). Rather, MHb nAChRs may be activated by an autocrine or paracrine ACh mechanism (Chung et al., 2023). MHb neurons release both ACh (Grady et al., 2009; Ren et al., 2011; Frahm et al., 2015) and glutamate (Ren et al., 2011; Frahm et al., 2015; Arvin et al., 2019) onto IPN neurons, though detectable levels of ACh can only be measured following strong stimulation of MHb axons (Ren et al., 2011; Souter et al., 2022). Beyond activating postsynaptic IPN nAChRs after release from synaptic terminals (Arvin et al., 2019), ACh may also be present in MHb neurons to facilitate efficient packaging of glutamate into synaptic vesicles (Frahm et al., 2015). Elimination of ACh synthesis in MHb ChAT-expressing neurons blocks nicotine conditioned place preference (Frahm et al., 2015). Interestingly, elimination of vesicular glutamate transporter 1 from the same MHb ChAT^+^ neurons blocks glutamate corelease from MHb axons and is associated with increased responding for nicotine in a self-administration assay (Souter et al., 2022). Cotransmission of ACh and glutamate in the MHb to IPN circuit is clearly important for normal neurophysiology and the behavioral response to nicotine.

Given the importance of glutamate release from MHb terminals onto IPN neurons (Souter et al., 2022) and given the importance of IPN neuron activity in a variety of behavioral and pharmacological responses (Zhao-Shea et al., 2013; Zhao-Shea et al., 2015; Molas et al., 2017; Klenowski et al., 2022; Klenowski et al., 2023; Molas et al., 2024), considering the specific role of glutamate receptors in IPN neurons is warranted. Transcriptional profiling of several IPN neuron subtypes indicates that IPN neurons may express ionotropic glutamate receptor (iGluR) subunits for α-amino-3-hydroxy-5-methyl-4-isoxazolepropionic acid (AMPA) and *N*-methyl-d-aspartic acid (NMDA) receptors (Ables et al., 2017). Mecamylamine-precipitated nicotine withdrawal boosts glutamate levels in IPN (Carcoba et al., 2022), and NMDA receptor (NMDAR) activity is required for IPN neuron activation and expression of somatic signs following this treatment (Zhao-Shea et al., 2013). iGluR function is plastic, as behavioral experience is sufficient to modulate AMPA receptor (AMPAR) activity (Kinoshita and Okamoto, 2023). IPN neurons expressing the α5 nAChR subunit may be particularly important for the response to acute and/or chronic nicotine. Aside from their expression of the α5 subunit, which is implicated in tobacco smoking and lung cancer (Bierut et al., 2008; Wang et al., 2009), α5^+^ neurons in the rostral IPN (IPR) subnucleus alter their expression of numerous signaling proteins in response to chronic nicotine exposure (Ables et al., 2017). It is not well understood, however, whether IPR neurons express functional iGluRs and, if so, whether their activity is modifiable by exposure to nicotine. Moreover, the subcellular distribution of functional iGluRs in IPN neurons has not been examined. In this study, we addressed these gaps using a combination of patch-clamp electrophysiology, two-photon imaging, glutamate uncaging, and nicotine intravenous self-administration (IVSA).

## Materials and Methods

### Materials

Injectable heparin sodium (catalog #00409-2720-02) was obtained from Pfizer (via Medline). Injectable meloxicam (catalog #07-893-7565) and isoflurane (catalog #07-894-8943) were obtained from Patterson Veterinary Supply. Nicotine hydrogen tartrate salt was obtained from Glentham Life Sciences (catalog #GL9693-5G). MNI-caged-l-glutamate, 6-cyano-7-nitroquinoxaline-2,3-dione (CNQX), d-(−)−2-amino-5-phosphonopentanoic acid (d-AP5), dihydro-β-erythroidine hydrobromide, methyllycaconitine citrate, SR 16584, tetrodotoxin citrate, picrotoxin, and QX314 chloride (QX314) were obtained from Tocris Bioscience. Atropine sulfate (atropine), mecamylamine hydrochloride, and dimethyl sulfoxide (DMSO) were obtained from Sigma-Aldrich.

### Animals

All experimental protocols involving vertebrates were reviewed and approved by the institutional Animal Care and Use Committee. Procedures also followed the guidelines for the care and use of animals provided by the National Institutes of Health Office of Laboratory Animal Welfare. All efforts were made to minimize animal distress and suffering during experimental procedures, including during the use of anesthesia. A total of *n* = 97 male SD rats (Envigo) were used. SD rats were ∼300 g (∼8 weeks old) when they arrived at our facility. Rats were housed at 22°C on a reverse 12/12 h light/dark cycle (4 P.M. lights on, 4 A.M. lights off).

### Operant chambers

Rats were trained in Med Associates operant chambers (interior dimensions, 11.9 × 9.4 × 11.3 in., catalog #MED-007-CT-B1) in sound-attenuating cubicles. The SA system was housed in a dedicated room within the same laboratory suite as the rat-housing room. A PC was used to control the SA system with a Med-PC IV software. Each chamber had transparent plastic walls and a stainless-steel grid floor and was equipped on the right-side wall with two nose pokes (2.4 in. from grid floor to nose poke center) flanking a pellet receptacle coupled to a pellet dispenser. A white stimulus light was located inside each nose poke with a house light located over the chamber's left-side wall. In all sessions, nose pokes on the active nose poke activated either the pellet dispenser or an infusion pump, respectively. Nose pokes on the inactive nose poke had no consequence. For intravenous drug infusions, each rat's catheter was connected to a liquid swivel via polyethylene tubing protected by a metal spring. The liquid swivel was connected to a 10 ml syringe loaded onto the syringe pump.

### Operant food training

Rats arrived pair housed and were only separated after surgery. A week after arrival, rats were food-restricted to enhance operant responding. Rats were fed standard chow (LabDiet Prolab RMH 3000 5P00, catalog #0001495), (20 g per rat, 40 g per cage) once per day at least 1 h after testing ended. Water was available *ad libitum* except during operant behavioral sessions. Food training sessions were 1 h in duration, and rats were trained to nose poke for food pellets (45 mg; Bio-Serv Dustless Precision Pellets, catalog #F0021) on the same nose poke hole that would later be paired with nicotine infusions. A fixed ratio 1 (FR1; no timeout) schedule was used for food training. No visual cues (stimulus, house light) were available during the session, and rats could earn a maximum of 75 food pellets during a 1 h session. Once 50 pellets with at least a 2:1 preference for the active nose poke relative to the inactive nose poke was achieved for two consecutive sessions, no further food training was conducted. These criteria were typically met after 3–5 d.

### Indwelling jugular catheter surgery

After acquiring food-operant responding, rats were anesthetized with isoflurane (3% induction, 2–3% maintenance) and implanted with indwelling jugular catheters (Instech, catalog #C30PU-RJV1402). Meloxicam (2 mg/kg) was administered immediately following surgery and for the next 2 d to reduce inflammation and pain. Rats were then singly housed and were given 7 d for recovery from surgery with catheters being flushed daily with heparin sodium dissolved in sterile saline.

### Intravenous drug self-administration

After recovery from catheter surgery, rats were allowed to self-administer saline or nicotine (0.03 mg/kg free base/infusion, volume ∼0.035 ml, 2.7–3.4 s infusion duration depending on body weight) during 2 h SA sessions, Monday through Friday (no SA sessions occurred on weekends). (−)-Nicotine hydrogen tartrate salt was dissolved in sterile saline, and the pH was adjusted to 7.4. Infusions, delivered by an infusion pump, were triggered by one nose poke response on the active nose poke. Infusions were simultaneously paired with illumination of the stimulus light over the active nose poke for 3 s. An active nose poke response that resulted in an infusion extinguished the house light for a 20 s timeout period (TO-20), during which responding was recorded but had no consequence. Responses on the inactive nose poke were recorded but had no scheduled consequence. At the end of the session, the house light was extinguished and responding had no consequences. Rats were removed from the training chambers as soon as possible after the end of the 2 h session and were rationed to 20 g of standard lab chow at least 1 h after finishing their sessions. Modified chow availability was used throughout self-administration and a range of 85–90% of free-feeding body weight was maintained. Rats were allowed to self-administer saline or nicotine for 10 sessions on a FR1/TO-20 schedule of reinforcement. If any one of the following occurred for nicotine self-administration, the rat did not move on to the intermittent access (IntA) portion of the study: (1) <10 nicotine infusions were earned within the 2 h session for two or more consecutive sessions, (2) the ratio of active to inactive nose pokes was <2.0 for three or more consecutive sessions, and (3) a drop of 75% or greater in responding on the active nose poke occurred over the final five sessions of acquisition. A total of two rats failed to meet the above criteria, with zero rats excluded due to loss of catheter patency. A total of *n* = 8 and *n* = 8 rats completed acquisition of nicotine IVSA and saline IVSA, respectively.

IntA procedures were based on previous publications (Zimmer and Roberts, 2012; Kawa et al., 2016; Tapia et al., 2022). Each session consisted of 12 alternating drug-available and no-drug-available periods lasting 5 and 15 min, respectively. The house light was on at the start of the session. The drug-available period started immediately after the rats were placed into the chamber. During the drug-available period, an active nose poke response (FR1/TO-20) resulted in an infusion, for which the house light was extinguished for a 20 s timeout period. During this time, responding was recorded but had no consequence. Responses on the inactive nose poke were recorded but had no scheduled consequence. After the 5 min period, the house light turned off and signaled a 15 min no-drug-available period. During the no-drug-available period, all nose pokes were recorded but had no consequences. The 5 min drug-available and 15 min no-drug-available periods repeated themselves for a total of 12 drug-available and 12 no-drug-available periods, resulting in a 4 h session, with 1 h of saline/nicotine availability. Rats self-administered saline/nicotine on the IntA paradigm for seven sessions.

### Brain slice preparation and recording solutions

Rats were anesthetized with isoflurane before trans-cardiac perfusion with oxygenated (95% O2/5% CO2), 4°C *N*-methyl-d-glucamine (NMDG)-based recovery solution that contains the following (in mM): 93 NMDG, 2.5 KCl, 1.2 NaH_2_PO_4_, 30 NaHCO_3_, 20 HEPES, 25 glucose, 5 sodium ascorbate, 2 thiourea, 3 sodium pyruvate, 10 MgSO_4_·7H2O, and 0.5 CaCl2·2H_2_O, 300–310 mOsm, pH 7.3–7.4. Brains were immediately dissected after the perfusion and held in oxygenated, 4°C recovery solution for 1 min before cutting a brain block containing the IPN and sectioning the brain with a vibratome (VT1200S; Leica). Coronal slices (250 µm) were sectioned through the IPN and transferred to oxygenated, 33°C recovery solution for 12 min. Slices were then kept in holding solution containing the following (in mM): 92 NaCl, 2.5 KCl, 1.2 NaH_2_PO_4_, 30 NaHCO_3_, 20 HEPES, 25 glucose, 5 sodium ascorbate, 2 thiourea, 3 sodium pyruvate, 2 MgSO_4_·7H_2_O, and 2 CaCl_2_·2H_2_O, 300–310 mOsm, pH 7.3–7.4, for 60 min or more before recordings. Brain slices were transferred to a recording chamber (1 ml volume), being continuously superfused at a rate of 1.5–2.0 ml/min with oxygenated 32°C recording solution. For our recording chamber and solution flow rate, we estimate that complete solution exchange occurs in 5–8 min. The recording solution contained the following (in mM): 124 NaCl, 2.5 KCl, 1.2 NaH_2_PO_4_, 24 NaHCO_3_, 12.5 glucose, 2 MgSO_4_·7H_2_O, 2 CaCl_2_·2H_2_O, 0.001 atropine sulfate, and 0.1 picrotoxin, 300–310 mOsm, pH 7.3–7.4. A Mg^2+^-free version of this recording solution was used where indicated in Results. Patch pipettes were pulled from borosilicate glass capillary tubes (1B150F-4; World Precision Instruments) using a programmable microelectrode puller (P-97; Sutter Instrument). Tip resistance ranged from 7.0 to 10.0 MΩ when filled with internal solution. A potassium gluconate-based internal solution was used for AMPAR recordings (in mM): 135 potassium gluconate, 5 EGTA, 0.5 CaCl_2_, 2 MgCl_2_, 10 HEPES, 2 MgATP, and 0.1 MgGTP, pH adjusted to 7.25 with Tris base, osmolarity adjusted to 290 mOsm with sucrose. NMDAR recordings employed the following internal solution (in mM): 117 CsCH_3_SO_3_, 0.4 EGTA, 2.8 NaCl, 20 HEPES, 5 TEA-Cl, 2.5 MgATP, and 0.25 MgGTP, pH adjusted to 7.25 with Tris base, osmolarity adjusted to 290 mOsm with sucrose. The internal solutions contained QX314 (2 mM) for improved voltage control.

### Two-photon laser scanning microscopy (2PLSM), electrophysiology, and glutamate uncaging

A modified Olympus BX51 upright microscope and a 60× (1.0 NA) water-dipping (2 mm working distance) objective were used to visualize cells. The Prairie View 5.5 (Bruker Nano) software was used for image acquisition, photostimulation, and electrophysiology acquisition via a Multiclamp 700B patch-clamp amplifier. Analog signals were sampled at 1 kHz, and an A/D converter (6,052; National Instruments) was used for digitization. Patch-clamp recordings were carried out using the internal solution mentioned above, except that Alexa Fluor 594 (hydrazide salt; 75 µM) was also included in the recording pipette to visualize cells using 2PLSM. Immediately prior to giga seal formation, the junction potential between the patch pipette and the superfusion medium was nulled. Series resistance was uncompensated. After break-in, the internal solution with the Alexa Fluor dye was allowed to equilibrate for 15–20 min before imaging was initiated. Cells were voltage-clamped at a holding potential of −60 mV unless otherwise indicated. A Chameleon Ultra I (Coherent Laser Group) tunable (690–1,040 nm) Ti:sapphire laser system tuned to 810 nm (80 MHz pulse repetition frequency and ∼140 fs pulse duration) was used to excite Alexa Fluor 594. A M350-80-02-BK Pockels cell (ConOptics) was used for Ti:sapphire laser power attenuation. The system was equipped with two nondescanned detectors (Hamamatsu side-on multialkali R3896 photomultiplier tubes) for detection of green and red wavelengths (emission filters, 525/70 nm, 595/50 nm), but only the red channel was used in this study. A 405 nm continuous wave laser (100 mW OBIS LX; Coherent) was used for photostimulation/uncaging via a partially independent light path and a second set of *x*–*y* galvanometers incorporated into the scanhead (Cambridge Technologies). MNI-glutamate (250 µM) was dissolved in 10 ml of recording solution, and the solution was applied to the slice via a recirculation system. The Markpoints module of the Prairie View 5.5 software was used to select spots in the field of view (∼1 µm diameter) for focal uncaging of glutamate via 405 nm laser light flashes (50 ms, 3–8 mW). For some recorded cells, a *Z*-series 2PLSM image of the cellular morphology was acquired after completion of electrophysiological recordings. A maximum intensity projection from such *Z*-series images was used to visualize cellular morphology in a single image.

### In situ hybridization and fluorescence microscopy

Rats were deeply anesthetized with isoflurane and decapitated. Brains were quickly removed on ice, snap frozen, and embedded in cryo-embedding medium (OCT). Brains were sectioned on a cryostat (CM3050; Leica) into 12 µm sections. Sections were adhered to Superfrost Plus slides and kept at −20°C to dry for 60 min and stored at −80°C until use. Sections were fixed with 4% paraformaldehyde and processed for RNAscope (Advanced Cell Diagnostics, ACD) multichannel fluorescent in situ hybridization according to the manufacturer manual for Multiplex assays. Sections were mounted with ProLong Gold Antifade Mountant with DAPI (Thermo Fisher Scientific). Probes for detection of specific targets (*Gria1*, *Grin1*, *Th*) were purchased from ACD (http://acdbio.com). Sections were imaged on an Olympus IX83 inverted microscope equipped with a 20× UPLXAPO objective (0.8 NA) and a 16 bit (2,048 × 2,048 pixel frame size) Hamamatsu ORCA-Flash 4.0 camera. Tiled, *z*-stack images were stitched together in the Olympus cellSens Dimension software and further processed with the ImageJ (NIH) and QuPath software. Briefly, *z*-stack images were processed with the cellSens Extended Focal Imaging (EFI) enhancement filter, which uses focus information from *z*-planes to construct a two-dimensional image that is optimally in focus. ImageJ was used to convert the EFI file to a TIF file. In QuPath, the Positive Cell Detection module was subsequently used to detect nuclei and identify cells that are positive and negative for each probe of interest. Intensity data for all detected cells were processed in Excel and GraphPad Prism.

### Experimental design and statistical analysis

The α level was set to 0.05 for all statistical tests, which were conducted with GraphPad Prism 10.6.1 software. Null hypothesis statistical testing was employed, where the null hypothesis stated, in general, that drug treatments have no effect on the physiological measures being taken. For perisomatic responses involving pharmacological treatments, a paired analysis approach (paired *t* test or repeated-measure one–way ANOVA with Tukey’s multiple-comparison testing) was used. For perisomatic responses comparing saline and nicotine IVSA, two approaches were used: (1) Responses in different cells from the same animal were used to compute a mean response for each animal, and the computed means for saline and nicotine IVSA rats represented each animal in an unpaired *t* test analysis. (2) Responses in multiple cells per animal (in saline and nicotine IVSA) were analyzed with a nested *t* test (mixed model). Analysis of electrophysiology data was performed with Clampfit (Molecular Devices) and/or custom scripts written in MATLAB (MathWorks).

## Results

**Figure 1.**
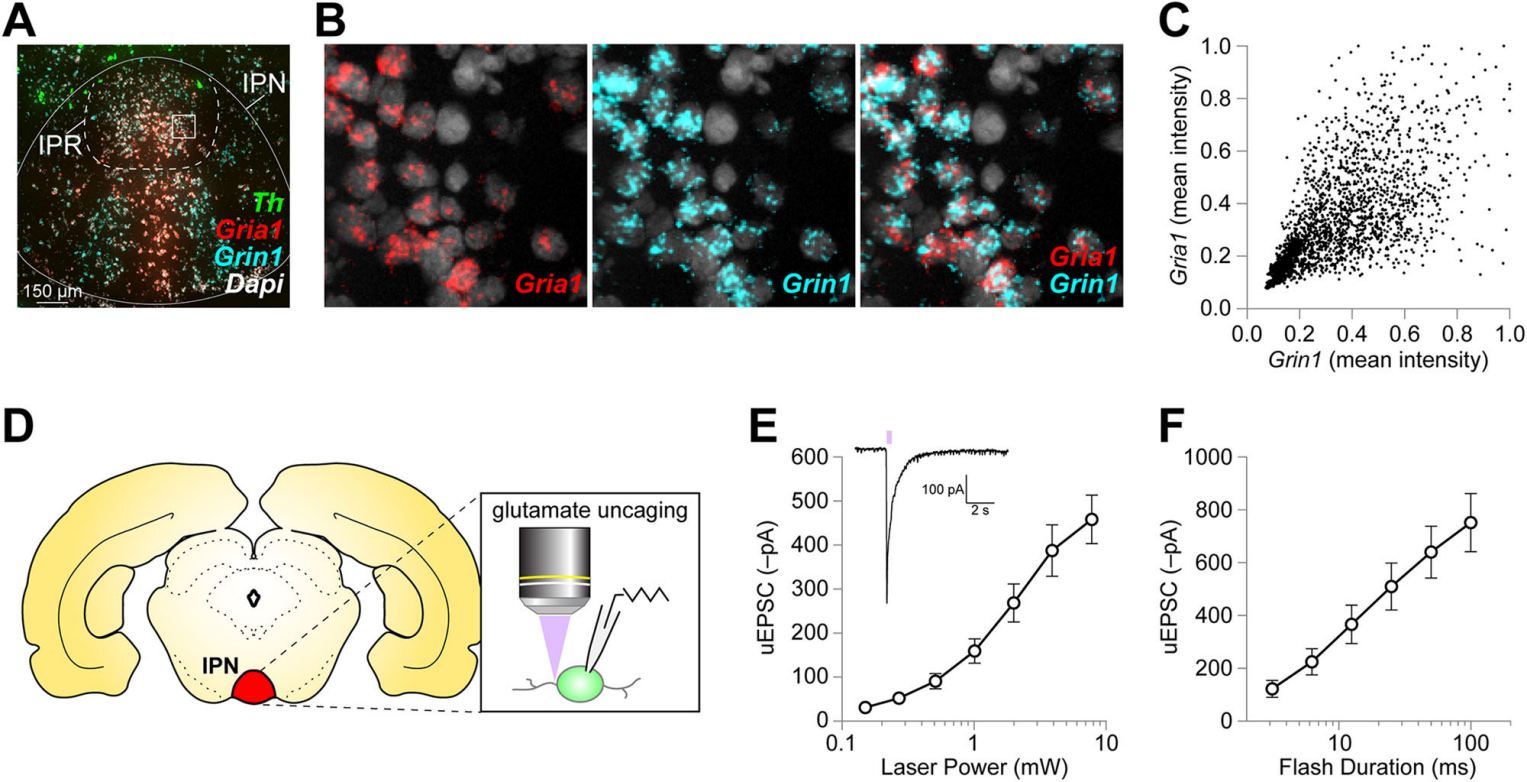
IPR neurons express iGluRs. ***A***, iGluR in situ hybridization. RNAscope in rat brain was conducted to detect the following targets: *Th*, *Gria1*, *Grin1*. ***A***, Representative fluorescence image is shown for a brain section containing the IPN with the indicated probes/colors. *Th* was used as a landmark to help identify the IPN. ***B***, *Gria1* and *Grin1* expression in IPR. The solid white boxed area from ***A*** shows IPR neurons, along with *Gria1* and *Grin1* expression therein. ***C***, *Gria1* and *Grin1* expression in IPR neurons. *Gria1* and *Grin1* expression in individual cells in the IPR, outlined in ***A***, was estimated and the two signals were plotted against each other for each cell. ***D***, Glutamate uncaging in IPN. The location of the IPN is shown in a rat brain section at the approximate bregma level where we conducted recordings. Slices at this bregma level were prepared, IPR neurons were patch clamped, and glutamate was uncaged via 405 nm laser stimulation at small (1–5 µm) spots near the recorded cell. ***E, F***, Glutamate uncaging photochemical dose–response. Glutamate was uncaged via a 50 ms flash of 405 nm light (***E***, inset). The laser power was systematically varied while keeping the flash duration constant, and the resulting uEPSC amplitude (mean ± SEM) is shown (***E***). Conversely, we modulated the flash duration while keeping the laser power constant (***F***). *n* = 4 brain slice/2 rats for ***C***; *n* = 29 locations/9 cells/5 rats for ***E***; and *n* = 26 location/9 cells/4 rats for ***F***.

We first examined iGluR expression in IPN by determining whether IPR neurons express mRNAs for the AMPAR and NMDAR subunits GluA1 (*Gria1*) and GluN1 (*Grin1*). Tyrosine hydroxylase (*Th*) mRNA, which marks nearby dopamine-producing neurons in VTA and SNc, was used as a landmark to help identify the IPN. We noted robust *Gria1* and *Grin1* expression in the IPN, with both showing expression in IPR (Fig. 1*A*,*B*). Additionally, *Grin1* appeared to be preferentially expressed in IPI, whereas *Gria1* was more preferentially expressed in IPC (Fig. 1*A*). For all IPR cells expressing either mRNA, we plotted *Grin1* versus *Gria1* fluorescence intensity. Both signals were correlated (*p* < 0.0001; Pearson's *r* = 0.6773) in the IPR (Fig. 1*C*; Table 1), generally supporting the idea that IPR neurons express iGluRs and receive glutamatergic input.

**Table 1.** Statistical table

Figure	Parameter	Sample size	Statistical test	Significance level
1*C*	*Grin1* versus *Gria1* mRNA expression	*n* = 2,384 cells from *n* = 2 rats	Pearson's correlation coefficient (r)	*p* < 0.0001; Pearson's *r* = 0.6773; *R*^2^ = 0.4587
3*C*	Uncaging current amplitude (−pA); perisoma; control versus 10 µM CNQX versus 50 µM CNQX	*n* = 7 cells from *n* = 6 rats	One-way repeated–measures ANOVA with Tukey’s post hoc multiple-comparison test	*p* < 0.0001 (ANOVA between columns; *F* = 138.8); *p* < 0.0001 (Tukey’s control versus CNQX 10 µM); *p* = 0.0034 (Tukey’s 10 µM CNQX versus 50 µM CNQX)
3*F*	Uncaging current amplitude (pA); perisoma; control versus d-AP5	*n* = 10 cells from *n* = 5 rats	Paired *t* test (two-tailed)	*p* = 0.0004; *t* = 5.377; df = 9
4*E*	Uncaging current amplitude (−pA); perisoma; saline SA versus nicotine SA	Saline: *n* = 28 cells from *n* = 8 rats Nicotine: *n* = 33 cells from *n* = 8 rats	Unpaired *t* test (two-tailed) on the computed mean current amplitude per rat, derived from multiple cells per rat	*p* = 0.018; *t* = 2.676; df = 14
4*F*	Uncaging current amplitude (−pA); perisoma; saline SA versus nicotine SA (same dataset as 4*E*)	Saline: *n* = 28 cells from *n* = 8 rats Nicotine: *n* = 33 cells from *n* = 8 rats	Nested *t* test (two-tailed, mixed model)	*p* = 0.015; *t* = 2.773; df = 14
4*G* (0.0 µM)	Uncaging current amplitude (−pA); perisoma; before nicotine versus after nicotine	*n* = 10 cells from *n* = 7 rats	Paired *t* test (two-tailed)	*p* = 0.071; *t* = 2.044; df = 9
4*G* (0.2 µM)	Uncaging current amplitude (−pA); perisoma; before nicotine versus after nicotine	*n* = 8 cells from *n* = 8 rats	Paired *t* test (two-tailed)	*p* = 0.405; *t* = 0.8858; df = 7
4*G* (0.6 µM)	Uncaging current amplitude (−pA); perisoma; before nicotine versus after nicotine	*n* = 8 cells from *n* = 7 rats	Paired *t* test (two-tailed)	*p* = 0.001; *t* = 5.244; df = 7
4*G* (1.8 µM)	Uncaging current amplitude (−pA); perisoma; before nicotine versus after nicotine	*n* = 8 cells from *n* = 7 rats	Paired *t* test (two-tailed)	*p* = 0.0002; *t* = 7.355; df = 7

Analysis details are shown for all figure panels with statistical analyses.

Next, we studied iGluR function in IPN with brain slice patch-clamp electrophysiology, two-photon laser scanning microscopy, and glutamate uncaging (Fig. 1*D*). After establishing whole-cell voltage–clamp recordings in IPR neurons, the cell was allowed to fill with Alexa Fluor 594 dye (provided via the patch pipette) for 20–30 min to enable visualization of cell morphology via two-photon imaging (810 nm imaging wavelength). MNI-glutamate (250 µM) was superfused, and glutamate was uncaged with a 405 nm laser. Relative to the recorded cell, the uncaging position was controlled via an independent *x*–*y* galvanometer system that allowed us to uncage glutamate at the soma or at any location in the dendritic arbor of the cell. Flashes of 405 nm light evoked robust inward currents in IPR neurons (Fig. 1*E*, inset) whose amplitude were sensitive to increases in laser power (Fig. 1*E*) and laser flash duration (Fig. 1*F*).

**Figure 2.**
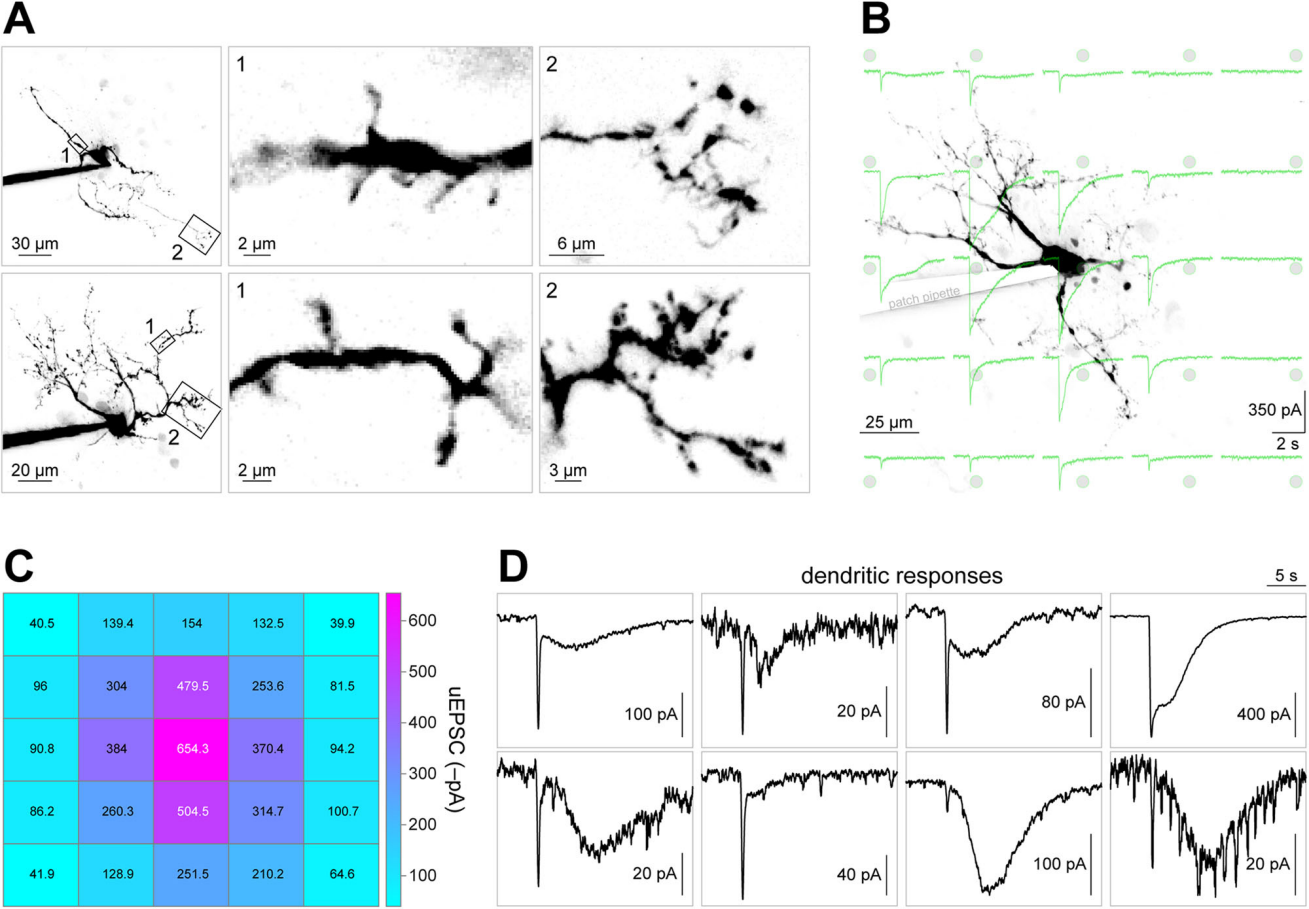
Glutamate uncaging at dendrites in IPR neurons. ***A***, Typical IPR neuron morphology. Two 2PLSM images (*z*-projection) of typical dye-filled IPR neurons are shown (left panels). Numbered (1 and 2) areas indicated with boxes are shown at higher resolution in the middle and right panels. These are representative of dendrites and dendritic spine morphology that we observed in rat IPR neurons. ***B***, Spatial uncaging in IPR neurons. A representative 2PLSM image is shown for an IPR neuron that was patch clamped (patch pipette is marked but covered for clarity) and filled with Alexa Fluor 594. A 5 × 5 square stimulation grid (gray spots with green outline), with 25 total uncaging locations, is shown. Inward iGluR response traces (green) at each location are shown. ***C***, Summary of spatial glutamate uncaging in IPR neurons. For *n* = 41 neurons analyzed as shown in ***B***, the mean response amplitude (denoted inside each location square) at each uncaging location is represented with a heat map. ***D***, Dendritic glutamate uncaging in IPR neurons. Representative dual-component responses from nonsomatic uncaging locations are shown. *n* = 1,025 locations/41 cells/23 rats for ***C***.

Next, we sought to determine whether IPR neurons express functional iGluRs on the surface of their dendrites. We examined IPR neurons via two-photon imaging of their morphology during patch-clamp recordings. IPR neurons often showed a modest-sized dendritic arbor, often with numerous short (2–5 µm) protrusions or spines from the main dendrite shaft (Fig. 2*A*). These spine-like protrusions are suggestive of synaptic contacts. To directly examine dendritic iGluR function, we designed a 5 × 5 *x*–*y* stimulation grid (25 uncaging locations) centered on the cell soma. A representative cell with this stimulation grid and the inward current response at each location is shown (Fig. 2*B*). For *n* = 41 cells analyzed in this manner, the mean inward current amplitude at each location is expressed as a heat map (Fig. 2*C*). iGluR responses at dendritic locations were quite variable. All responses had a fast inward component that activated and decayed (or desensitized) within ∼10–50 ms. Often, these iGluR responses had a second, slower component with variable (1) amplitude, (2) latency to onset, and (3) decay time (Fig. 2*D*).

**Figure 3.**
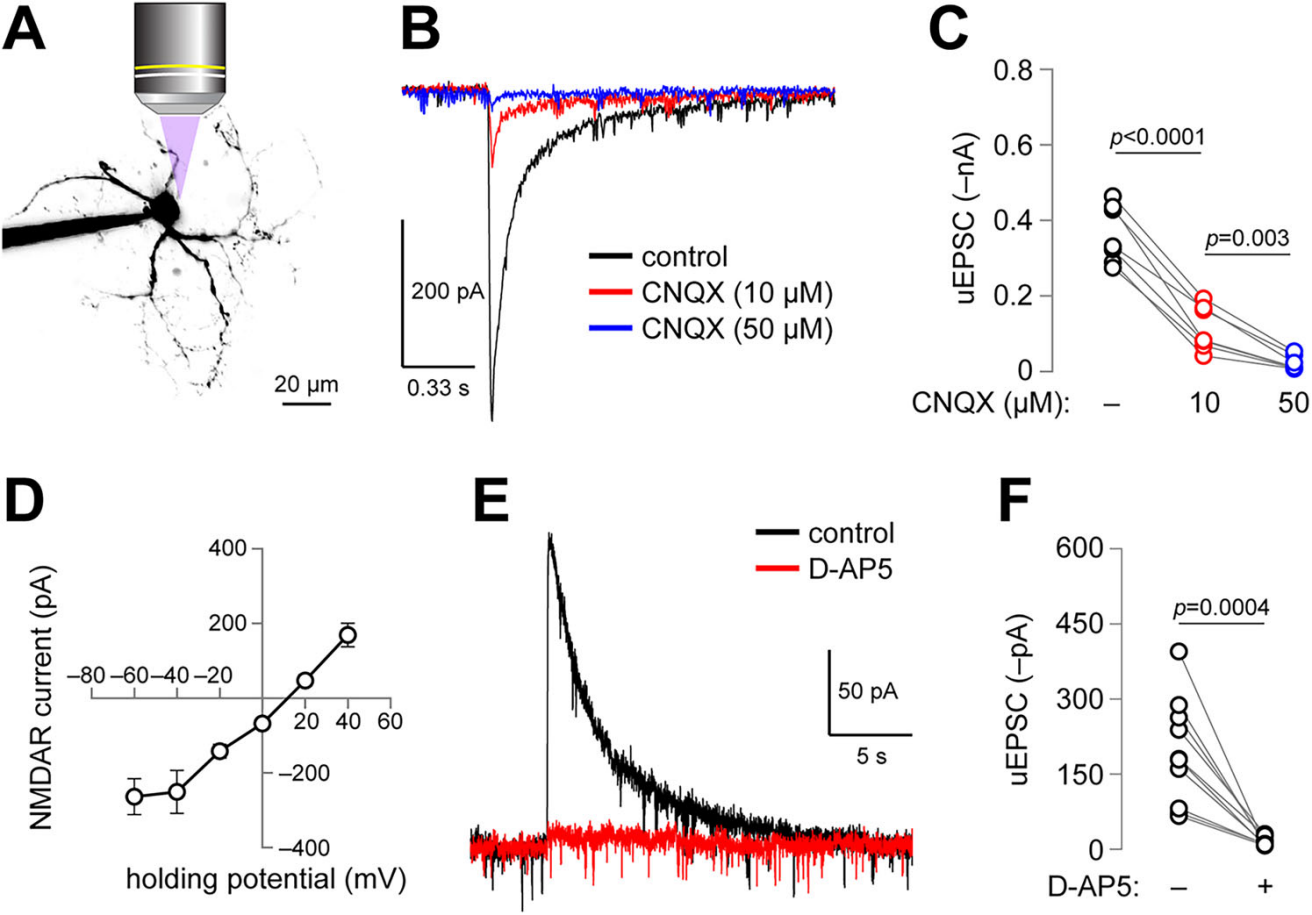
Perisomatic iGluR function in IPR neurons. ***A***, Representative IPR neuron. A 2PLSM image (*z*-projection) of a dye-filled IPR neuron is shown, with an uncaging schematic to indicate the typical perisomatic uncaging location. ***B***, CNQX-sensitivity of perisomatic uncaging responses. Perisomatic uncaging responses from a representative cell, held at −60 mV, is shown under control conditions (d-AP5 treated) and in response to 10 µM CNQX, then 50 µM CNQX. ***C***, A before–after plot for the effect of CNQX (10 µM then 50 µM) on perisomatic uncaging responses in IPR neurons. ***D***, Current–voltage relation for NMDAR currents in IPR neurons. ***E***, d-AP5 sensitivity of perisomatic uncaging responses. Perisomatic uncaging responses from a representative cell, held at +40 mV, is shown under control conditions and in response to d-AP5. ***F***, A before–after plot for the effect of d-AP5 on perisomatic uncaging responses in IPR neurons. *n* = 7 cells from *n* = 6 rats for ***C***, *n* = 12 cells from *n* = 5 rats for ***D***, and *n* = 10 cells from *n* = 5 rats for ***F***.

To identify the iGluRs in our recordings, we uncaged glutamate at the soma of IPR neurons before and after pharmacological antagonism of AMPAR or NMDAR. A two-photon image of a representative IPR neuron is shown, including the approximate location of the perisomatic uncaging location (Fig. 3*A*). To isolate putative AMPARs, d-AP5 was applied to the slice, and glutamate was uncaged while holding the cell at −60 mV (Fig. 3*B*; control). CNQX (10 µM) blocked a majority of the inward current, but a residual current remained (Fig. 3*B*,*C*). When we applied a higher CNQX concentration (50 µM), the residual current was more fully blocked (Fig. 3*B*,*C*; Table 1). Next, we isolated NMDAR currents via CNQX pretreatment and a Mg^2+^-free recording solution to alleviate Mg^2+^-associated NMDA channel block. A cesium-based internal solution was used to facilitate recordings at depolarized holding potentials. Under these conditions, glutamate-evoked currents exhibited a fairly linear current–voltage relation that is characteristic of NMDARs when free of Mg^2+^-block (Nowak et al., 1984; Fig. 3*D*). d-AP5 treatment fully blocked glutamate uncaging-evoked currents under these conditions (Fig. 3*E*,*F*; Table 1). Together, these data confirm functional expression of AMPAR and NMDAR in IPR neurons.

**Figure 4.**
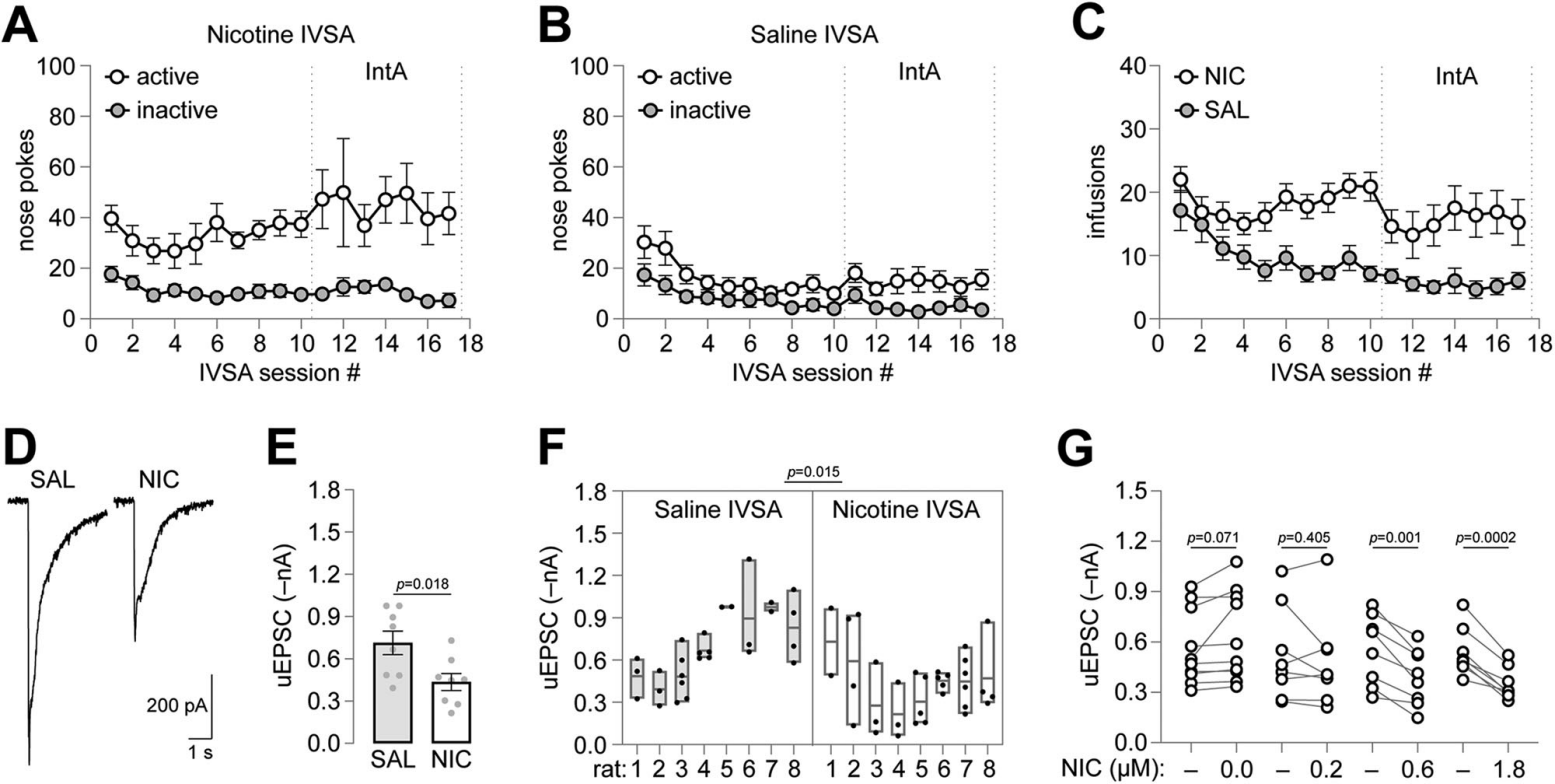
Nicotine self-administration and acute exposure reduces iGluR responses in IPR. ***A–C***, Active and inactive nose pokes (***A***, nicotine IVSA; ***B***, saline IVSA) and nicotine or saline infusions (***C***) are shown for 17 IVSA sessions. Sessions 1–10 (2 h FR1) and 11–17 (IntA) are denoted. ***D***, Nicotine IVSA reduces perisomatic glutamate uncaging response amplitude in IPR. Representative responses for perisomatic glutamate uncaging in IPR neurons is shown for saline IVSA and nicotine IVSA rats. ***E, F*** Summary data for perisomatic glutamate uncaging amplitude in saline and nicotine IVSA. Data points in ***E*** represent the average response for all cells from the same animal. An unpaired *t* test *p* value is shown. Data in ***F*** show individual cells for each rat using a box plot. A nested *t* test *p* value is shown. ***G***, Summary nicotine concentration–response. iGluR response amplitude is shown before and after superfusion of the indicated nicotine concentration. Each pair represents an independent experiment on one IPR cell in a slice; slices were discarded after treatment with only one nicotine concentration. *n* = 8 rats per group for ***A***–**C**. For ***E***–***F***, *n* = 28 cells from *n* = 8 saline IVSA rats and *n* = 33 cells from *n* = 8 nicotine IVSA rats. For ***G***, *n* = 10 cells from *n* = 7 rats for 0.0 µM nicotine; *n* = 8 cells from *n* = 8 rats for 0.2 µM nicotine; *n* = 8 cells from *n* = 7 rats for 0.6 µM nicotine; *n* = 8 cells from *n* = 7 rats for 1.8 µM nicotine.

To further examine the significance of iGluR responses in IPR neurons, we asked whether exposure to nicotine could modulate them. Male SD rats were implanted with indwelling jugular catheters and were trained to self-administer saline or nicotine for 17 sessions. Sessions 1–10 were 2 h FR1 sessions, and Sessions 11–17 were IntA sessions. Male rats quickly develop a preference for the active nose poke compared with the inactive nose poke (Fig. 4*A*). As expected, active responses for saline infusions declined over the course of the SA sessions. Self-infusions were steady at ∼20/session (Fig. 4*C*), which equates to a total exposure of ∼0.6 mg/kg. Immediately after Session 17, rats were killed, and brain slices were prepared for patch-clamp recordings in IPR. Compared with saline IVSA, perisomatic responses from IPR neurons held at −60 mV showed a reduction in amplitude for rats with experience self-administering nicotine (Fig. 4*D*). Perisomatic glutamate uncaging currents from multiple cells per animal were used to compute a mean response amplitude for each of *n* = 8 saline IVSA and *n* = 8 nicotine IVSA animals. Glutamate currents were significantly reduced in nicotine IVSA animals compared with saline IVSA (Fig. 4*E*; Table 1). The same glutamate current data were analyzed with a nested *t* test (mixed model), which also confirmed a significant reduction in amplitude in nicotine IVSA animals versus saline (Fig. 4*F*; Table 1). Next, we asked whether acute nicotine could reduce iGluR currents in a manner similar to nicotine IVSA. IPR-containing slices were prepared from rats that did not undergo any drug treatment. Uncaging-evoked iGluR currents were recorded before and after bath application of vehicle or only one of three nicotine concentrations (in µM): 0.2, 0.6, and 1.8. Vehicle treatment served as a control for any drift in iGluR currents over time. Indeed, there was a weak trend toward increased iGluR current over time, as revealed by vehicle treatment (Fig. 4*G*; Table 1). Nicotine (0.6 and 1.8 µM) treatment caused a significant reduction in iGluR current amplitude, whereas 0.2 µM nicotine treatment did not cause a significant change (Fig. 4*G*; Table 1). These data corroborate our findings from IVSA animals, indicating that nicotine can acutely modulate iGluRs in IPR neurons.

## Discussion

In this study, we report a characterization of iGluR function, pharmacology, and information about their activity in cellular compartments in rat IPR neurons. These cells robustly express AMPA and NMDA subunit mRNA transcripts and functional receptors at the cell soma. IPR neurons possess dendritic spine-like structures, and we find a wide variety of responses to uncaged glutamate at dendritic locations. Nicotine exposure, either to slices from naive animals or via nicotine self-administration behavioral assays, downregulates iGluR function in IPR neurons. Overall, iGluR function in IPR neurons is robust and modifiable in response to nicotine.

Our demonstration of the existence of functional iGluRs in IPR is consistent with previous reports showing spontaneous and/or evoked excitatory postsynaptic currents (EPSCs) in IPN neurons. McGehee, Role, and colleagues recorded CNQX-sensitive EPSCs at habenulo→interpeduncular synapses in a cell culture system (McGehee et al., 1995). Other publications (Ren et al., 2011; Hsu et al., 2013; Frahm et al., 2015; Arvin et al., 2019) have previously reported spontaneous and evoked EPSCs in IPN neurons that are sensitive to traditional AMPA or NMDAR blockers. Spontaneous EPSCs in IPR neurons were recently shown to be modulated by mu opioid receptor activation (Singhal et al., 2025). Although a prior study previously noted two cell populations in mouse IPR that can be differentiated by their electrophysiological response to locally applied AMPA and nicotine (Shih et al., 2014), we did not find a similar dual population in our rat experiments with MNI-glutamate uncaging. Whereas 10 µM CNQX is typically used to fully block AMPARs, 50 µM was needed to fully block these receptors in our experiments (Fig. 3*C*).

IPN iGluR activity plays a role in some important behavioral processes. Notably, nicotine withdrawal is associated with increased frequency of EPSCs in IPN (Zhao-Shea et al., 2013), and the NMDAR antagonist d-AP5 is sufficient to block nicotine withdrawal signs when injected in the IPN prior to mecamylamine-precipitated withdrawal induction (Zhao-Shea et al., 2013). Mice with disrupted glutamate transmission in IPN have altered locomotor responses to nicotine, blunted nicotine withdrawal, and reduced nicotine place preference behavior (Frahm et al., 2015).

IPN neurons are morphologically heterogeneous. A previous study using two-photon imaging of dye-filled, patch-clamped IPN neurons indicated substantial variability in dendritic complexity among these cells (Arvin et al., 2019). Consistent with iGluR responses noted above, some IPN neurons were found to possess dendritic protrusions that appear similar to dendritic spines (Arvin et al., 2019). These were found on cells with both elaborate and sparse dendritic arbors (Arvin et al., 2019). The current study corroborates these findings; IPR neurons were identified with extensive dendritic arbors, often with spine-like protrusions (Fig. 2*A*). Interestingly, neurons of the lateral subnucleus of the IPN (IPL) have low-complexity dendritic arbors and lack these protrusions/spines (Jin and Drenan, 2022). IPN neurons are known to develop specialized synaptic contact points called crest synapses (Murray et al., 1979), which make a synaptic connection with axons from both hemispheres of the MHb (Lenn et al., 1983). In a future study, it would be worthwhile to determine whether the dendritic spines we imaged are related to crest synapses.

iGluR responses are reduced in IPR neurons of rats with a history of nicotine self-administration. Our experiment with acute nicotine application to slices from rats naive to nicotine corroborates our results from IVSA rats, suggesting that nicotine downregulates iGluR responses after even a brief treatment. These results indicate that brief exposure to nicotine may dampen IPR neuron sensitivity to incoming glutamatergic input from MHb axons. In the absence of nicotine, ongoing acetylcholine release from MHb axons may play a role in iGluR responses (Ren et al., 2011). The IPN is a brain area with dense cholinergic fibers (Banala et al., 2018) and nAChR-stimulated ACh release (Grady et al., 2001; Grady et al., 2009). This area may have unusually strong coupling between nicotinic cholinergic signaling and homeostatic modulation of iGluR functional activity.

Our study has limitations. First, only male rats were studied. Future studies should include experiments in female rats. Second, MNI-glutamate uncaging supplies ectopic glutamate to the slice preparation. Although our laser photostimulation approach is much more precise than glutamate superfusion to the slice, we cannot rule out the possibility that glutamate was applied at nonphysiological concentrations and/or that glutamate was applied for a nonphysiological duration of time.

The IPN, and especially the IPR (Banala et al., 2018), is composed primarily of GABAergic neurons. This includes projection neurons sending afferents to targets such as the raphe and laterodorsal tegmental nucleus (Quina et al., 2017; Wolfman et al., 2018), along with parvalbumin (PV)- and somatostatin (SST)-expressing interneurons (Zhao-Shea et al., 2013; Arvin et al., 2019). In IPR neurons, reduced iGluR responsiveness following nicotine exposure may lead to reduced excitability and therefore less action potential firing and less GABA release from IPR neuron axons in downstream structures. IPR projections to laterodorsal tegmentum influence VTA neuron activity. One or more targets of IPR neurons may be disinhibited by nicotine exposure via this mechanism. Our results show that IPR neuron sensitivity to glutamate may be dynamically modulated by nicotine. This is consistent with the MHb and IPN's important role in mediating aspects of somatic and affective signs of nicotine withdrawal in animal models. Modulated glutamate sensitivity in IPR neurons may play a role in human tobacco dependence.

## Synthesis

Reviewing Editor: Christie Fowler, University of California Irvine Decisions are customarily a result of the Reviewing Editor and the peer reviewers coming together and discussing their recommendations until a consensus is reached. When revisions are invited, a fact-based synthesis statement explaining their decision and outlining what is needed to prepare a revision will be listed below. The following reviewer(s) agreed to reveal their identity: NONE.

This manuscript expands our understanding of ionotropic glutamate transmission from the MHb to the IPN in the context of nicotine self-administration, and demonstrates that ACh enhances response to glutamate via agonism, rather than via desensitization, of nAChRs. Both reviewers appreciated the revisions and felt the manuscript has been improved to support the conclusions.

Minor comment: Line 29: "modulate the excitability of IPN neurons" is the excitability measured here? You measured excitatory synaptic transmission.

Congratulations!

## References

[B1] Ables JL, et al. (2017) Retrograde inhibition by a specific subset of interpeduncular α5 nicotinic neurons regulates nicotine preference. Proc Natl Acad Sci U S A 114:13012–13017. 10.1073/pnas.1717506114 29158387 PMC5724287

[B2] Aizawa H, Kobayashi M, Tanaka S, Fukai T, Okamoto H (2012) Molecular characterization of the subnuclei in rat habenula. J Comp Neurol 520:4051–4066. 10.1002/cne.23167 22700183

[B3] Arvin MC, Jin XT, Yan Y, Wang Y, Ramsey MD, Kim VJ, Beckley NA, Henry BA, Drenan RM (2019) Chronic nicotine exposure alters the neurophysiology of habenulo-interpeduncular circuitry. J Neurosci 39:4268–4281. 10.1523/JNEUROSCI.2816-18.2019 30867261 PMC6538858

[B4] Banala S, et al. (2018) Photoactivatable drugs for nicotinic optopharmacology. Nat Methods 15:347–350. 10.1038/nmeth.4637 29578537 PMC5923430

[B5] Bierut LJ, et al. (2008) Variants in nicotinic receptors and risk for nicotine dependence. Am J Psychiatry 165:1163–1171. 10.1176/appi.ajp.2008.07111711 18519524 PMC2574742

[B6] Carcoba LM, Uribe KP, Ortegon S, Mendez IA, DeBiasi M, O’Dell LE (2022) Amino acid systems in the interpeduncular nucleus are altered in a sex-dependent manner during nicotine withdrawal. J Neurosci Res 100:1573–1584. 10.1002/jnr.24826 33751631 PMC8455708

[B7] Chung L, Jing M, Li Y, Tapper AR (2023) Feed-forward activation of habenula cholinergic neurons by local acetylcholine. Neuroscience 529:172–182. 10.1016/j.neuroscience.2023.07.030 37572877 PMC10840387

[B8] Forget B, et al. (2018) A human polymorphism in CHRNA5 is linked to relapse to nicotine seeking in transgenic rats. Curr Biol 28:3244–3253 e3247. 10.1016/j.cub.2018.08.044 30293722

[B9] Fowler CD, Lu Q, Johnson PM, Marks MJ, Kenny PJ (2011) Habenular α5 nicotinic receptor subunit signalling controls nicotine intake. Nature 471:597–601. 10.1038/nature09797 21278726 PMC3079537

[B10] Frahm S, et al. (2011) Aversion to nicotine is regulated by the balanced activity of β4 and α5 nicotinic receptor subunits in the medial habenula. Neuron 70:522–535. 10.1016/j.neuron.2011.04.013 21555077

[B11] Frahm S, Antolin-Fontes B, Gorlich A, Zander JF, Ahnert-Hilger G, Ibanez-Tallon I (2015) An essential role of acetylcholine-glutamate synergy at habenular synapses in nicotine dependence. eLife 4:e11396. 10.7554/eLife.11396 26623516 PMC4718731

[B12] Grady SR, Meinerz NM, Cao J, Reynolds AM, Picciotto MR, Changeux JP, McIntosh JM, Marks MJ, Collins AC (2001) Nicotinic agonists stimulate acetylcholine release from mouse interpeduncular nucleus: a function mediated by a different nAChR than dopamine release from striatum. J Neurochem 76:258–268. 10.1046/j.1471-4159.2001.00019.x 11145999

[B13] Grady SR, Moretti M, Zoli M, Marks MJ, Zanardi A, Pucci L, Clementi F, Gotti C (2009) Rodent habenulo-interpeduncular pathway expresses a large variety of uncommon nAChR subtypes, but only the α3β4* and α3β3β4* subtypes mediate acetylcholine release. J Neurosci 29:2272–2282. 10.1523/JNEUROSCI.5121-08.2009 19228980 PMC2680386

[B14] Hsu YW, Tempest L, Quina LA, Wei AD, Zeng H, Turner EE (2013) Medial habenula output circuit mediated by α5 nicotinic receptor-expressing GABAergic neurons in the interpeduncular nucleus. J Neurosci 33:18022–18035. 10.1523/JNEUROSCI.2927-13.2013 24227714 PMC3828458

[B15] Jin XT, Drenan RM (2022) Functional alpha7 nicotinic acetylcholine receptors in GABAergic neurons of the interpeduncular nucleus. Neuropharmacology 208:108987. 10.1016/j.neuropharm.2022.108987 35167902 PMC8885883

[B16] Kawa AB, Bentzley BS, Robinson TE (2016) Less is more: prolonged intermittent access cocaine self-administration produces incentive-sensitization and addiction-like behavior. Psychopharmacology (Berl) 233:3587–3602. 10.1007/s00213-016-4393-8 27481050 PMC5023484

[B17] Kinoshita M, Okamoto H (2023) Acetylcholine potentiates glutamate transmission from the habenula to the interpeduncular nucleus in losers of social conflict. Curr Biol 33:2121–2135 e2124. 10.1016/j.cub.2023.03.087 37105168

[B18] Klenowski PM, Zhao-Shea R, Freels TG, Molas S, Tapper AR (2022) Dynamic activity of interpeduncular nucleus GABAergic neurons controls expression of nicotine withdrawal in male mice. Neuropsychopharmacology 47:641–651. 10.1038/s41386-021-01107-1 34326477 PMC8782840

[B19] Klenowski PM, Zhao-Shea R, Freels TG, Molas S, Zinter M, M’Angale P, Xiao C, Martinez-Nunez L, Thomson T, Tapper AR (2023) A neuronal coping mechanism linking stress-induced anxiety to motivation for reward. Sci Adv 9:eadh9620. 10.1126/sciadv.adh9620 38055830 PMC10699782

[B20] Lenn NJ, Wong V, Hamill GS (1983) Left-right pairing at the crest synapses of rat interpeduncular nucleus. Neuroscience 9:383–389. 10.1016/0306-4522(83)90301-9 6877600

[B21] McGehee DS, Heath MJ, Gelber S, Devay P, Role LW (1995) Nicotine enhancement of fast excitatory synaptic transmission in CNS by presynaptic receptors. Science 269:1692–1696. 10.1126/science.7569895 7569895

[B22] Molas S, Zhao-Shea R, Liu L, DeGroot SR, Gardner PD, Tapper AR (2017) A circuit-based mechanism underlying familiarity signaling and the preference for novelty. Nat Neurosci 20:1260–1268. 10.1038/nn.4607 28714952 PMC5752132

[B23] Molas S, Freels TG, Zhao-Shea R, Lee T, Gimenez-Gomez P, Barbini M, Martin GE, Tapper AR (2024) Dopamine control of social novelty preference is constrained by an interpeduncular-tegmentum circuit. Nat Commun 15:2891. 10.1038/s41467-024-47255-y 38570514 PMC10991551

[B24] Murray M, Zimmer J, Raisman G (1979) Quantitative electron microscopic evidence for reinnervation in the adult rat interpeduncular nucleus after lesions of the fasciculus retroflexus. J Comp Neurol 187:447–468. 10.1002/cne.901870211 489788

[B25] Nowak L, Bregestovski P, Ascher P, Herbet A, Prochiantz A (1984) Magnesium gates glutamate-activated channels in mouse central neurones. Nature 307:462–465. 10.1038/307462a0 6320006

[B26] Perry DC, Xiao Y, Nguyen HN, Musachio JL, Davila-Garcia MI, Kellar KJ (2002) Measuring nicotinic receptors with characteristics of α4β2, α3β2 and α3β4 subtypes in rat tissues by autoradiography. J Neurochem 82:468–481. 10.1046/j.1471-4159.2002.00951.x 12153472

[B27] Quick MW, Ceballos RM, Kasten M, McIntosh JM, Lester RA (1999) Α3β4 subunit-containing nicotinic receptors dominate function in rat medial habenula neurons. Neuropharmacology 38:769–783. 10.1016/S0028-3908(99)00024-6 10465681

[B28] Quina LA, Harris J, Zeng H, Turner EE (2017) Specific connections of the interpeduncular subnuclei reveal distinct components of the habenulopeduncular pathway. J Comp Neurol 525:2632–2656. 10.1002/cne.24221 28387937 PMC5873981

[B29] Ren J, Qin C, Hu F, Tan J, Qiu L, Zhao S, Feng G, Luo M (2011) Habenula “cholinergic” neurons co-release glutamate and acetylcholine and activate postsynaptic neurons via distinct transmission modes. Neuron 69:445–452. 10.1016/j.neuron.2010.12.038 21315256

[B30] Salas R, Pieri F, De Biasi M (2004) Decreased signs of nicotine withdrawal in mice null for the β4 nicotinic acetylcholine receptor subunit. J Neurosci 24:10035–10039. 10.1523/JNEUROSCI.1939-04.2004 15537871 PMC6730195

[B31] Sheffield EB, Quick MW, Lester RA (2000) Nicotinic acetylcholine receptor subunit mRNA expression and channel function in medial habenula neurons. Neuropharmacology 39:2591–2603. 10.1016/S0028-3908(00)00138-6 11044729

[B32] Shih PY, Engle SE, Oh G, Deshpande P, Puskar NL, Lester HA, Drenan RM (2014) Differential expression and function of nicotinic acetylcholine receptors in subdivisions of medial habenula. J Neurosci 34:9789–9802. 10.1523/JNEUROSCI.0476-14.2014 25031416 PMC4099552

[B33] Singhal SM, Szlaga A, Chen YC, Conrad WS, Hnasko TS (2025) Mu-opioid receptor activation potentiates excitatory transmission at the habenulo-peduncular synapse. Cell Rep 44:115874. 10.1016/j.celrep.2025.115874 40540395 PMC12352575

[B34] Souter EA, Chen YC, Zell V, Lallai V, Steinkellner T, Conrad WS, Wisden W, Harris KD, Fowler CD, Hnasko TS (2022) Disruption of VGLUT1 in cholinergic medial habenula projections increases nicotine self-administration. eNeuro 9:ENEURO.0481-21.2021. 10.1523/ENEURO.0481-21.2021 PMC8751853 34876472

[B35] Tapia MA, et al. (2022) Relapse-like behavior and nAChR sensitization following intermittent access nicotine self-administration. Neuropharmacology 212:109066. 10.1016/j.neuropharm.2022.109066 35461879 PMC9527938

[B36] Wang JC, et al. (2009) Genetic variation in the CHRNA5 gene affects mRNA levels and is associated with risk for alcohol dependence. Mol Psychiatry 14:501–510. 10.1038/mp.2008.42 18414406 PMC4381434

[B37] Wolfman SL, Gill DF, Bogdanic F, Long K, Al-Hasani R, McCall JG, Bruchas MR, McGehee DS (2018) Nicotine aversion is mediated by GABAergic interpeduncular nucleus inputs to laterodorsal tegmentum. Nat Commun 9:2710. 10.1038/s41467-018-04654-2 30006624 PMC6045623

[B38] Zhao-Shea R, Liu L, Pang X, Gardner PD, Tapper AR (2013) Activation of GABAergic neurons in the interpeduncular nucleus triggers physical nicotine withdrawal symptoms. Curr Biol 23:2327–2335. 10.1016/j.cub.2013.09.041 24239118 PMC3855889

[B39] Zhao-Shea R, et al. (2015) Increased CRF signalling in a ventral tegmental area-interpeduncular nucleus-medial habenula circuit induces anxiety during nicotine withdrawal. Nat Commun 6:6770. 10.1038/ncomms7770 25898242 PMC4405813

[B40] Zimmer BA, Roberts DC (2012) Cocaine self-administration in rats: hold-down procedures. Methods Mol Biol 829:279–290. 10.1007/978-1-61779-458-2_18 22231821

